# Out-of-pocket expenditure for seeking health care for sick children younger than 5 years of age in Bangladesh: findings from cross-sectional surveys, 2009 and 2012

**DOI:** 10.1186/s41043-017-0110-4

**Published:** 2017-09-11

**Authors:** Tazeen Tahsina, Nazia Binte Ali, D.M. Emdadul Hoque, Tanvir M. Huda, Shumona Sharmin Salam, Mohammad Mehedi Hasan, Md Altaf Hossain, Ziaul Matin, Lianne Kuppen, Sarah P. Garnett, Shams El Arifeen

**Affiliations:** 1 Maternal and Child Health Division (MCHD), icddr,b, Dhaka, Bangladesh; 2 IMCI, Directorate General of Health Services (DGHS), Ministry of Health and Family Welfare (MOHFW), Dhaka, Bangladesh; 3 Health Section, UNICEF Bangladesh, Dhaka, Bangladesh; 4 The Children’s Hospital at Westmead Clinical School, University of Sydney, Westmead, NSW Australia

**Keywords:** Out-of-pocket expenditure, Childhood illnesses, Under-five children, Health care-seeking, Bangladesh

## Abstract

**Background:**

Bangladesh has committed to universal health coverage, and options to decrease household out-of-pocket expenditure (OPE) are being explored. Understanding the determinants of OPE is an essential step. This study aimed to estimate and identify determinants of OPE in seeking health care for sick under-five children.

**Methods:**

Cross-sectional data was collected by structured questionnaire in 2009 (*n* = 7362) and 2012 (*n* = 6896) from mothers of the under-five children. OPE included consultation fees and costs of medicine, diagnostic tests, hospital admission, transport, accommodation, and food. Expenditure is expressed in US dollars and adjusted for inflation. Linear regression was used for ascertaining the determinants of OPE.

**Results:**

Between 2009 and 2012, the median OPE for seeking care for a sick under-five child increased by ~ 50%, from USD 0.82 (interquartile range 0.39–1.49) to USD 1.22 (0.63–2.36) per child/visit. Increases were observed in every component OPE measured, except for consultation fees which decreased by 12%. Medicine contributed the major portion of overall OPE. Higher overall OPE for care seeking was associated with a priority illness (20% increase), care from trained providers (90% public/~ 2-fold private), residing in hilly/wet lands areas (20%), and for mothers with a secondary education (19%).

**Conclusion:**

OPE is a major barrier to quality health care services and access to appropriate medicine is increasing in rural Bangladesh. To support the goal of universal health care coverage, geographic imbalances as well as expanded health financing options need to be explored.

**Electronic supplementary material:**

The online version of this article (10.1186/s41043-017-0110-4) contains supplementary material, which is available to authorized users.

## Background

Global mortality in children under 5 years of age (under-five children) has declined by over 50% in the last 25 years [1]. Consistent with this trend, Bangladesh has decreased under-five mortality from 133 deaths per 1000 live births in 1993 to 38 deaths per 1000 live births in 2015 [2]. However, under-five mortality in Bangladesh is still high, approximately four times that in the WHO European Region and almost double the Sustainable Development Goal of 25 deaths per 1000 births in 2030 [1, 3]. The situation is worse in rural areas where mortality rates are higher compared to urban areas (55 vs 50 in 2014) [2]. The leading causes of mortality are acute respiratory infections including pneumonia, serious infections, and birth asphyxia [1]. Seeking appropriate care by trained providers in adequately equipped health care facilities is critical for improving health outcomes and reducing mortality [4]. Yet, health care-seeking behavior for a sick under-five child in Bangladesh is low (~ 30%) and there are substantial health inequalities; the poor are less likely to seek care for their under-five child, less likely to use trained health care providers, and child mortality rate is twice as high in the poorest families compared to the wealthiest families [5, 6].

Out-of-pocket expenditure (OPE), which includes consultation fees, cost of medicine, and transport, required at the time of treatment is a major barrier to seeking health care [7, 8]. Bangladesh has one of the lowest total health expenditures as a proportion of gross domestic product in South Asia, 3.5% in 2007, and households bear the majority of health expenditure [9]. In 1997, households accounted for 57% of total health expenditure which increased to 63% in 2011 [10]. Recent research found OPE to have significant association with age, sex, marital status, place of residence, and household wealth status among all population of Bangladesh [11]. In urban Bangladesh, OPE and illness-related loss of income have been associated with the sale of assets, debt, and reduction in essential consumption resulting in financial catastrophe [12]. Similar outcomes have been reported in families of children with pneumonia admitted into a large pediatric hospital in Dhaka [13]. No prior literature was found with estimates of OPE for care-seeking for under-five illnesses in rural Bangladesh. There is a paucity of data on OPE for seeking health care for under-five sick children in rural Bangladesh. In 2011, the Government of Bangladesh made a commitment to universal health coverage by 2032. That is, all people will have access to the health care they need without suffering financial hardship. As part of this process, options to mobilize additional financial resources and decrease OPE, particularly for the poor, are being explored [14]. Understanding the current determinants of OPE in rural areas for illness among children under 5 years of age is an important step. Hence, the objective of this study was to estimate OPE and identify determinants of OPE in seeking health care for sick under-five children living in rural Bangladesh and see whether there was any effect of Maternal, Newborn and Child Survival (MNCS) intervention on OPE.

## Methods

The Maternal, Newborn and Child Survival (MNCS) program was a cluster randomized control trial designed to determine the effectiveness of community-based interventions in improving selected child care practices and child nutrition with the overall aim of decreasing child morbidity and mortality in Bangladesh. The MNCS program was aimed at women with under-five children living in five rural districts, Bandarban, Gopalganj, Kishoreganj, Netrokona, and Sunamganj, with high infant morbidity and mortality, between June 2009 and March 2012. The intervention included an antenatal and postnatal care package providing information on the best maternal care practices including recognizing danger signs and was delivered by trained health providers. Evaluation of the program followed a cluster randomized control trial design. The clusters were selected following probability proportionate to size (PPS) sampling method and assigned to intervention and comparison. Each district had both intervention and comparison clusters.

Data for this study was collected by household surveys at commencement (2009) and endpoint (2012) of the MNCS program. At the study commencement and endpoint, a total of 546 villages were randomly selected, out of 2411 villages in the five districts. From each village, 34 households were selected for interview. In 2009, a total of 10,239 eligible women aged 15–49 years were interviewed which included 7501 mothers of under-five children. The response rate was 99.9%. Of the 7501 mothers, 7362 (98.2%) completed the OPE questionnaire covering 7490 under-five children. In 2012, 10,455 eligible women were interviewed of which 6925 had under-five children and the response rate was 99.8%. Of the 6925 mothers, 6896 (99.6%) completed the OPE questionnaire having 6944 under-five children.

### Ethics, consent and permissions

The ethics review board of icddr,b approved the study, and the study conformed to the principles embodied in the Declaration of Helsinki (GR 00699). Informed consent was sought before enrollment. Written consent was obtained from literate participants and a thumb print from illiterate participants.

### Household survey

Household surveys were carried out from July to September 2009 and February to March 2012. A pretested structured questionnaire was used for data collection at both time points. Mothers with under-five children were asked about sociodemographic characteristics, including age, marital status, education, and wealth. Education was stratified into five categories: no education, primary incomplete, primary complete, secondary incomplete, and secondary or higher. Wealth was determined by ownership of household durable goods, dwelling characteristics, source of drinking water, sanitation facility, possession of land, and domestic animals using principle component analysis. Sources of drinking water were categorized into improved source (pipe water, tube well, or protected dug well) and unimproved source (ponds, river, unprotected dug well). Improved source of sanitation facilities included flush latrine or pit latrine, whereas unimproved source sanitation facilities comprised of pit latrine without slab or no facilities at all. Wealth was stratified into five quintiles where “one” represented the poorest and “five” represented the richest. The environment where the mothers lived was documented and categorized as plain, wet, or hilly land. Wet or hilly lands were categorized as hard-to-reach areas. The mothers were also asked about the prevalence of illness in the under-five child in the 2 weeks preceding the interview, associated care-seeking and cost of care-seeking. For example, mothers were asked if their child had had any of the following symptoms: fever, convulsion, cough, sneezing, runny nose, chest in drawing, stridor, body rash, loose bowel motions, vomiting, earache, unable to eat/drink, and yellow coloration of the body. If any of these symptoms were reported, mothers were asked where they sought care and from who. Health care providers were categorized into public (trained provider working in the government health care facilities), private trained (trained provider working in private health care facilities), health providers from NGOs, private untrained (informal health care providers including pharmacy, ayurveda, homeopath, and traditional healer), and village doctors (little or no training). Information on expenditure incurred for care-seeking was collected for up to four subsequent visits. Components of OPE covered in the survey included expenditure for both patients and their accompanying caregivers. Illnesses were categorized into priority illness (children who had pneumonia, diarrhea with dehydration, dysentery, malaria, or measles) and non-priority illness (children who had a cough or cold, acute or chronic ear infection, diarrhea with no dehydration, anemia, or fever without malaria) [15].

### Out-of-pocket expenditure on health care for under-five sick child

OPE included both direct and indirect costs. The direct costs were consultancy fees, medicine costs, diagnostic, and hospital admission costs. Indirect costs included transport to and from the health provider, food during visits, and hotel/accommodation costs if families/patients had stay overnight. Mothers could report on up to four consecutive visits and on more than one under-five children, if relevant. Expenditure was collected in Bangladesh taka and converted to US dollars (USD), with a conversion rate of 1 USD = 77.7 taka. Both for 2009 and 2012, the data were adjusted for inflation using the national consumer price index with 2005–2006 as the base year.

### Data analysis

Data was analyzed using Stata 12 Special Edition software. Mean OPE was calculated by aggregating direct costs and indirect costs per child per visit. For univariate analysis, median costs are presented. Kruskal-Wallis tests were used to examine the differences in median OPE and expenditure on medicine between groups. Explanatory variables for regression analysis were selected based on a priori knowledge and from the findings of bivariate analysis. Linear regression was used to explore the determinants of OPE and expenditure on medicine. OPE was not normally distributed and was log transformed where the zero costs turned into missing values. For the categorical explanatory variables, dummy variables were used. The overall intention of the analysis was not to look at the intervention effect; however, the intervention group was used as a covariate in the analysis in order to adjust any indirect effect on Out of Pocket Expenditure (OPE). Positive changes in care-seeking between baseline and end line period may have caused increase in OPE over time. An interaction variable has been added for adjusting intervention and time effect. The final model included all the significant (*P* < 0.05) explanatory variables and other relevant variables based on literature. The assumptions of modeling (normality and multicollinearity [VIF < 2]) were tested and met.

## Results

Mothers of 7490 and 6944 under-five children were surveyed in 2009 and 2012, respectively (Table 1). At both time points, the mean age of the women was 28 years, most (98%) were married, and ~ 40% had no formal education. At both time periods, there was approximately the same number of boys and girls and > 80% were aged 12 months or older (Table 2). In 2009, 50% of women reported that their child had an illness in the 2 weeks preceding the survey and health care was sought for over 70%, of which 14% was from a trained provider. In 2012, 34% of women reported that their children had an illness in the 2 weeks preceding the survey and health care was sought for 83%, of which 21% was from a trained provider.

**Table 1:** Sociodemographic distribution of mother of under-five children expressed in percentage in 2009 and 2012

Characteristics	2009	2012
Women (*n*)	7362	6896
Age (years)		
15–19	3.3	4.3
20–24	28.2	27.7
25–29	31.9	30.9
30–34	18.1	20.1
35–39	11.8	10.5
40–49	6.8	6.5
Marital status		
Currently married	98.4	98.4
Education		
No education	44.3	40.8
Primary incomplete (1–4 years)	20.0	17.6
Primary complete (5 years)	13.7	16.8
Secondary incomplete (6–9 years)	17.5	20.3
Secondary or higher (10+ years)	4.5	4.5
Wealth		
Poorest	20.6	19.9
Second	20.0	20.8
Middle	20.5	20.6
Fourth	19.3	19.6
Richest	19.6	19.1

**Table 2:** Demographic characteristics, morbidity, and care-seeking of under-five children expressed in percentage in 2009 and 2012

Characteristics	2009	2012
Children (*n*)	7490	6944
Sex (boys)	50.8	51.9
Age (months)		
< 6	7.3	8.7
6–11	11.0	9.7
12–23	18.4	16.4
24–35	21.3	21.2
36–47	22.1	21.0
48–59	20.0	22.9
Any illness in the last 2 weeks	50.4	34.3
Non-priority illness^1^	73.6	77.6
Priority illness^2^	26.4	22.2
Care sought from provider	71.8	82.5
Care sought from trained health care provider^3^	13.6	20.7
Location of household		
Plain land	33.1	55.1
Hard-to-reach areas	66.8	44.5
Area		
Comparison	50.9	50.1
Intervention	49.1	49.9

^1^Non-priority illness: children who had a cough or cold, acute or chronic ear infection, diarrhea with no dehydration, anemia, or fever without malaria

^2^Priority illness: children who had pneumonia, diarrhea with dehydration, dysentery, malaria, or measles

^3^Trained provider working in public or private health care facilities

OPE for seeking care for under-five children in 2009 and 2012 are shown in Table 3. At both time periods, expenditure on medicine constituted the highest proportion of OPE. Between 2009 and 2012, the total median OPE expenditure for seeking care for a sick under-five child increased by ~ 50% after adjusting for inflation, from USD 0.82 to USD 1.22 per child/visit. Increases were observed in every component of direct and indirect expenditure measured, except for consultation fees which decreased by 12%. A 2.5-fold increase in expenditure was observed on diagnostic tests per child/visit, and expenditure on hospital admission increased by 2-fold per child/visit.

**Table 3:** Median out-of-pocket expenditure for seeking care for under-five children in 2009 and 2012

	2009^1^		2012^1^	
	Median USD (*n*)^2^	IQR^2^	Median USD (*n*)^2^	IQR^2^
Total cost	0.82 (3009)	(0.39–1.49)	1.22 (2318)	(0.63–2.36)
Direct costs	0.68 (2916)	(0.34–1.21)	0.93 (2274)	(0.50–1.68)
Consultation fees	0.48 (925)	(0.19–0.72)	0.42 (997)	(0.34–0.84)
Medicine	0.58 (2822)	(0.29–1.00)	0.72 (2184)	(0.39–1.26)
Diagnostic	0.19 (182)	(0.13–0.48)	0.67 (87)	(0.25–1.68)
Hospital admission	0.14 (25)	(0.10–0.38)	0.42 (51)	(0.21–1.68)
Indirect costs	0.24 (1682)	(0.11–0.53)	0.50 (1297)	(0.25–1.18)
Transport	0.21 (1626)	(0.10–0.48)	0.50 (1273)	(0.25–1.01)
Accommodation	1.26 (30)	(0.19–2.88)	2.58 (33)	(1.26–5.05)
Food	0.19 (225)	(0.14–0.48)	0.42 (232)	(0.25–0.84)

^1^Adjusted for inflation

^2^Median (*IQR* interquartile range) expenditure is expressed in US dollars (USD) per child/visit

In both 2009 and 2012, median OPE was higher for priority illness compared to non-priority illness, Table 4. Those who sought care from trained providers (public and private) and lived in hard-to-reach areas also incurred higher costs for care seeking. Mothers with more years of education also tended to have higher OPE. There was no statistical significant difference between intervention and comparison areas in terms of OPE.

**Table 4:** Median out-of-pocket expenditure and expenditure on medicine by type of illness, provider, and socio-demographic characteristics

	Total OPE^1^		Expenditure on medicine^1^	
Indicator	2009^2^	2012^2^	2009^2^	2012^2^
Any illness	0.82	1.22	0.58	0.72
Non-priority^3^	0.77	1.16	0.53	0.69
Priority^4^	0.96	1.49	0.67	0.76
Health care provider				
Untrained	0.75	1.03	0.56	0.65
Public trained^5^	1.21	2.31	0.82	1.10
Private trained^5^	1.95	2.94	0.92	1.09
Non-government organization	0.48	0.68	0.29	0.36
Mother’s education				
No education	0.77	1.17	0.58	0.67
Primary incomplete (1–4 years)	0.77	1.17	0.55	0.73
Primary complete (5 years)	0.78	1.18	0.57	0.72
Secondary incomplete (6–9 years)	0.97	1.30	0.62	0.69
Secondary complete or higher (10+ years)	1.06	1.45	0.71	0.93
Wealth				
Poorest	0.85	1.18	0.58	0.72
Second	0.85	1.26	0.61	0.67
Middle	0.77	1.22	0.51	0.70
Fourth	0.78	1.22	0.58	0.76
Highest	0.81	1.24	0.62	0.67
Location of household				
Plain land	0.77	1.14	0.58	0.67
Hard-to-reach areas	0.85	1.29	0.59	0.76
Area				
Comparison	0.82	1.22	0.60	0.67
Intervention	0.85	1.22	0.58	0.72

^1^Median expenditure is expressed in US dollars (USD) per child per visit

^2^Adjusted for inflation

^3^Non-priority illness: children who had a cough or cold, acute or chronic ear infection, diarrhea with no dehydration, anemia, or fever without malaria

^4^Priority illness: children who had pneumonia, diarrhea with dehydration, dysentery, malaria, or measles

^5^Trained provider working in public or private health care facilities

Table 5 illustrated the demographic characteristics, morbidity, and care-seeking pattern for zero cost of care-seeking in 2009 and 2012. Majority of the mothers who did not incur any cost for care-seeking for under-five illnesses had no educational attainment. Mothers whose children suffered from non-priority illnesses mostly incurred zero cost for care-seeking at both point of time. In 2009, around 72% participants with zero cost for care-seeking sought care from untrained provider while in 2012, around 60% sought care from public trained providers or NGOs.

**Table 5:** Demographic characteristics, morbidity, and care-seeking of under-five children expressed in percentage in 2009 and 2012 with zero cost

Characteristics	2009 *n* = 158	2012 *n* = 108
Type of illnesses		
Non-priority illness^1^	76.0	82.4
Priority illness^2^	24.1	17.6
Care sought from provider		
Untrained	72.1	37.0
Public trained	25.3	49.1
Private trained	2.0	2.8
Non-government organization	0.5	11.1
Mother’s education		
No education	48.7	43.5
Primary incomplete (1–4 years)	18.9	16.7
Primary complete (5 years)	9.5	18.5
Secondary incomplete (6–9 years)	17.1	15.7
Secondary or higher (10+ years)	5.7	5.6
Wealth		
Poorest	19.6	25.0
Second	20.2	25.0
Middle	20.2	14.8
Fourth	15.2	21.3
Richest	24.7	13.8
Location of household		
Plain land	46.2	66.4
Hard-to-reach areas	53.8	33.6
Area		
Comparison	34.8	25.9
Intervention	65.1	74.0

^1^Non-priority illness: children who had a cough or cold, acute or chronic ear infection, diarrhea with no dehydration, anemia, or fever without malaria

^2^Priority illness: children who had pneumonia, diarrhea with dehydration, dysentery, malaria, or measles

To determine the best predictive model for OPE, the data from the 2009 and 2012 surveys were combined, Table 6. Mothers who had a child with a priority illness incurred nearly 20% higher overall OPE compared to children with non-priority illness. OPE for care-seeking from private trained providers was nearly two fold higher than seeking care from untrained provider. Children whose mothers had at least commenced secondary education spent 19% more than mothers with no education. Our analysis also indicates that people living in hard-to-reach areas (hilly and wetlands) had a 20% higher OPE compared to those living in plain lands. For OPE on medicine, mothers of children with priority illness spent 12% higher compared to those with non-priority illness. Those who sought care from private trained providers incurred 70% higher OPE on medicine, while mothers with secondary or higher education incurred 16% higher, and those living in hard-to-reach areas incurred 10% higher OPE on medicine compared to mothers with no education and those living in plain land. Household wealth status was not significantly associated with total OPE and expenditure on medicine.

**Table 6:** Determinants of out-of-pocket expenditure and expenditure on medicine for seeking care for under-five children

	Total OPE^1^		Expenditure on medicine^1^	
	Coefficient	*P* value	Coefficient	*P* value
Illness type				
Non-priority^2^	Ref		Ref	
Priority^3^	0.20	< 0.000	0.12	< 0.000
Provider type				
Untrained	Ref		Ref	
Public trained	0.90	< 0.000	0.48	< 0.000
Private trained	1.77	< 0.000	0.70	< 0.000
Non-government organization	− 0.24	0.157	− 0.30	0.061
Mother’s education				
No education	Ref		Ref	
Primary incomplete (1–4 years)	− 0.01	0.719	0.01	0.806
Primary complete (5 years)	0.05	0.218	0.03	0.391
Secondary incomplete (6–9 years)	0.19	< 0.000	0.06	0.101
Secondary complete or higher (10+ years)	0.19	< 0.05	0.16	< 0.05
Wealth quintiles				
Poorest	Ref		Ref	
Second	− 0.01	0.817	0.01	0.810
Middle	− 0.05	0.215	− 0.02	0.592
Fourth	− 0.04	0.327	0.01	0.801
Highest	− 0.08	0.079	− 0.02	0.678
Location of household				
Plain land	Ref		Ref	
Hard-to-reach areas	0.20	< 0.000	0.10	< 0.001
Increase of expenditure from 2009 to 2012				
2009	Ref		Ref	
2012	0.85	< 0.000	0.22	< 0.000
Area				
Comparison	Ref		Ref	
Intervention	0.06	0.090	0.01	0.854
Interaction of area and time	− 0.06	0.304	0.09	0.091

^1^The 2009 and 2012 expenditure have been combined

^2^Non-priority illness: children who had a cough or cold, acute or chronic ear infection, diarrhea with no dehydration, anemia, or fever without malaria

^3^Priority illness: children who had pneumonia, diarrhea with dehydration, dysentery, malaria, or measles

## Discussion

Between 2009 and 2012, OPE in seeking care for under-five children living in five rural districts of Bangladesh increased by almost 50% after adjusting for inflation. While there was an increase in all items measured, medicine was the major contributor of OPE followed by transport. The results also indicate that the OPE was higher for children with a priority illness, if care was sought from a trained provider, for more educated mothers and if the household was located in a hard-to-reach area. Wealth was not associated with OPE, and the poorest had a similar OPE to the least poor.

The results from our study suggest the previous reported trend in increasing OPE per capita between 2000 and 2010 is continuing. Data from the Bangladesh national Household Income and Expenditure Surveys (HIES) demonstrated that OPE per capita for health increased by 30% from 2000 to 2005 and doubled from 2005 to 2010. Between 1997 and 2007, OPE grew at 14% annually, faster than the annual growth rate of GDP (10%) [16]. The growing reliance on OPE leaves the population at risk of increased morbidity and mortality and/or financial catastrophe. Recent studies in urban Bangladesh estimated that between 9 and 18% of households faced financial catastrophe when seeking health care and that the poorest households had a four times higher risk of catastrophe than the least poor households [17, 18]. On a positive note, cause for higher OPE could be that in developing countries higher income is associated with more intensive use of health services in both private and public sectors. Improved education levels, especially of women, and more even distribution and control of resources within the household between the men and women can also lead to higher spending on necessary health services that were previously not utilized [19–21].

In the MNCS study areas, overall care-seeking from both public and private trained providers increased between the two survey periods. For priority illness, care-seeking from trained providers increased from 24.6 to 30.8%. As a result, OOP increased from USD 2.1 to USD 3.4 between baseline to end line for priority illness. Interestingly, hospital admission between baseline to end line also increased despite a fall in priority illness. There has also been a threefold increase in severe diseases among under-two children coupled with increased admission of under-two children from 2 to 4% between the two survey periods (Additional file 1: Tables S1 and S2). All these may be possible reasons triggering a higher OOP. Our analysis looking at the characteristics of the participants with zero cost for care-seeking also confirms that mothers having no educational attainment and who sought care from untrained provider, public provider, or NGOs mostly incurred no cost for care-seeking. An alternative interpretation of the high level of OPE in the MNCS area is that people are still able to bear the costs of the health care, even if they are causing financial strain. The resulting effect of poor people forgoing health care because of unaffordable costs on their health and subsequent earnings could have longer-term implications for poverty rates [22].Vouchers and/or cash incentives (demand supply financing) for mothers seeking care for their under-five children has the potential to decrease OPE as well as increase use of the formal health care system. This approach has been shown to increase the number of births in health facilities in Bangladesh, however, had it had no impact on the overall equity of use; poor patients were less likely to take advantage of the opportunity [23].

Consistent with previous studies in Bangladesh examining OPE for seeking care at maternal and child health services [5] and overall health care [13, 16], medicine was the key contributor to OPE for seeking care for under-five children. Medicine is purchased to self-treat, or purchased on the advice of a trained (public and private) or informal health care providers, or after consulting drug sellers (most are not qualified) at pharmacies. Overall, between 2009 and 2012 OPE on medicine increased by 24% in real terms. In part, this may be a result of increased use of trained providers between 2009 and 2012 as well as an increase in price, but the increase may also be due to changing prescription patterns. Over prescription of medicine (including antibiotics), multi-medicine prescriptions, and prescribing unnecessary expensive medicines have been previously reported in Bangladesh [24].

It is also interesting to note that expenditure on medicine as a proportion of OPE decreased from 59% in 2009 to 46% in 2012. A trend which had been previously reported using the Ministry of Health and Family Welfare 2010 data, which documented a decrease in OPE medicine on overall health from 74% in 1997 to 63% in 2007 [16]. Nevertheless, the cost of medicine is the main reason for high cost of visits to government facilities [16]and is a bigger burden for the poor and contributes to the inequalities in health. OPE could be considerably reduced by increasing supply of free medicine in public facilities.

OPE was higher if the household was located in a hard-to-reach areas and transport accounted for a substantial percentage of OPE, 21% in 2009 and 29% in 2012. Overall travel costs per capita have been previously described as very low (~ 1% per capita) [5]. The discrepancy, at least in part, probably demonstrates the increased expenditure for those living in rural area and hard-to-reach areas. Disparities in health care services between urban and rural areas and hard-to-reach areas of the country have been well documented in Bangladesh. The discrepancies have been recognized by the Government of Bangladesh, and one of the aims in the current health sector program (Health, Population and Nutrition Sector Development Program 2011–2016) is to incentivize service delivery in hard-to-reach areas. Additional workforce or establishment of mobile facilities or virtual consultations may help reduce regional disparities in care-seeking and expenditure related to it. In addition to OPE, other barriers to seeking care from trained providers also need to be acknowledged. These include preference for local pharmacies and village doctors [2], in contrast to facility-based care, are frequently known to the mothers, are readily available and may have a flexible payment system [16].

The strength of this study was the large sample size of randomly selected participants living in rural area with high maternal and child morbidity and mortality. However, there were a number of limitations. Data for this study was obtained from two cross-sectional surveys conducted as part of the evaluation of MNCS program. Information regarding OPE over time and its determinants were collected as part of secondary objective of the original evaluation to look at cost effectiveness of the intervention. Ideally, a time series dataset would have been more appropriate to depict change in OPE over time. However, this was not the primary objective, and we do not see any significant change in the quality of our analysis where we adjusted for time as a determinant of OPE. Additionally, the data are likely to underestimate the burden of illness as information regarding opportunity cost of illness was not collected. Opportunity costs include income loss or time cost of the caregiver that is incurred by the household during the period of illness of their children. There is a paucity of data relating to opportunity cost of seeking health care in developing countries. However, there is one study undertaken in rural Burkina Faso, West Africa which indicates that OPE may be higher than 70% of total expenditure [25].

## Conclusion

This study demonstrates that OPE is increasing in five districts in rural Bangladesh, which have been identified as having high infant morbidity and mortality. High OPE is a major barrier to quality health care services and access to appropriate and affordable medicine. Further research is needed to explore the pricing mechanisms of essential medicine as well as consumer preferences for purchasing and provider preferences prescribing medicine. To support the government’s goal of universal health care coverage by 2032, geographic imbalances as well as expanded health financing options should be explored such as government payments, incentives, and/or health insurance. With the new sector plan to be rolled out this year and having one prime component to deliver an Essential Service Package for improving universal health coverage, our findings can help in refining and verifying the national plan of action and national child health strategy.

## Additional file

Additional file 1: Supplementary Tables.
**Table S1**. Care-seeking from different types of providers for priority and non-priority illnesses. **Table S2**. OPE and drug cost across different types of providers for priority and non-priority illnesses (excluding zero cost). (DOCX 13 kb)

## Abbreviations

HIES: National Household Income and Expenditure Surveys
MNCS: Maternal, Newborn and Child Survival
OPE: Out-of-pocket expenditure
