# Supplementary material for: Out-of-pocket expenditure for seeking health care for sick children younger than 5 years of age in Bangladesh: findings from cross-sectional surveys, 2009 and 2012
Source: J Health Popul Nutr. 2017 Sep 11;36:33. doi: 10.1186/s41043-017-0110-4 (PMC5594455; doi:10.1186/s41043-017-0110-4)
Supplement: Additional file 1: Supplementary Tables. — Table S1. Care-seeking from different types of providers for priority and non-priority illnesses. Table S2. OPE and drug cost across different types of providers for priority and non-priority illnesses (excluding zero cost). (DOCX 13 kb) [file 41043_2017_110_MOESM1_ESM.docx]

**Table S1: Care seeking from different types of providers for priority and non-priority illnesses:**

|  | **2009** | | **2112** | |
| --- | --- | --- | --- | --- |
|  | **Priority illness**  **%(n)** | **Non-Priority illness %(n)** | **Priority illness %(n)** | **Non-Priority illness %(n)** |
| Untrained | 75.0(625) | 82.4(1924) | 68.2(371) | 75.8(1427) |
| Public trained^4^ | 18.0(150) | 11.3(264) | 22.2(121) | 15.6(294) |
| Private trained^4^ | 6.6(55) | 5.7( 133) | 8.6(47) | 7.6(142) |
| Non- government organization | 0.3(3) | 0.6(13) | 0.9(5) | 1.0(19) |

**Table S2: OPE and drug cost across different types of providers for priority and non-priority illnesses (excluding zero cost):**

| **Total OPE** | **Priority illness (OPE)** | | **Non-Priority illness (OPE)** | |
| --- | --- | --- | --- | --- |
|  | **2009** | **2012** | **2009** | **2112** |
| Overall | 0.96 | 1.48 | 0.77 | 1.16 |
| Untrained | 0.86 | 1.10 | 0.71 | 1.01 |
| Public trained | 1.25 | 2.61 | 1.13 | 2.10 |
| Private trained | 2.12 | 3.36 | 1.86 | 2.83 |
| Non- government organization | 0.48 | 0.15 | 0.76 | 1.36 |
| **Expenditure on medicine** | **Priority illness (Drug cost)** | | **Non-Priority illness (Drug cost)** | |
|  | **2009** | **2012** | **2009** | **2112** |
| Overall | 0.67 | 0.75 | 0.55 | 0.69 |
| Untrained | 0.67 | 0.64 | 0.50 | 0.65 |
| Public trained | 0.86 | 1.10 | 0.78 | 1.10 |
| Private trained | 0.99 | 1.39 | 0.91 | 0.95 |
| Non-government organization | 0.48 | 0.13 | 0.25 | 0.37 |
